# The role of adipogenic niche resident cells in colorectal cancer progression in relation to obesity

**DOI:** 10.1111/obr.13873

**Published:** 2025-01-06

**Authors:** Mikołaj Domagalski, Joanna Olszańska, Katarzyna Pietraszek‐Gremplewicz, Dorota Nowak

**Affiliations:** ^1^ Department of Cell Pathology, Faculty of Biotechnology University of Wroclaw Wroclaw Poland

**Keywords:** adipocytes, adipose tissue, colorectal cancer, obesity

## Abstract

Colorectal cancer (CRC) is the third most common cancer worldwide and has one of the highest mortality rates. Considering its nonlinear etiology, many risk factors are associated with CRC formation and development, with obesity at the forefront. Obesity is regarded as one of the key environmental risk determinants for the pathogenesis of CRC. Excessive food intake and a sedentary lifestyle, together with genetic predispositions, lead to the overgrowth of adipose tissue along with a disruption in the number and function of its building cells. Adipose tissue‐resident cells may constitute part of the CRC microenvironment. Alterations in their physiology and secretory profiles observed in obesity may further contribute to CRC progression, and despite similar localization, their contributions are not equivalent. They can interact with CRC cells, either directly or indirectly, influencing various processes that contribute to tumorigenesis. The main aim of this review is to provide insights into the diversity of adipose tissue resident cells, namely, adipocytes, adipose stromal cells, and immunological cells, regarding the role of particular cell types in co‐forming the CRC microenvironment. The scope of this study was also devoted to the abnormalities in adipose tissue physiology observed in obesity states and their impact on CRC development.

## 1 INTRODUCTION

The tumor microenvironment (TME) is a leading research topic in tumor biology. In recent decades, its components have been identified as essential for cancer development and metastasis.^1^ The composition of the TME exhibits notable variability depending on the type of tumor and surrounding cells. Colorectal cancer (CRC) is a representative example, and the pivotal role of the TME has been established.^1^, ^2^ Nevertheless, due to the intricacy of the interactions, the holistic role of the TME in this tumor eludes a clear definition.

Despite extensive research on the biology of CRC, the mechanisms underlying its formation and metastasis remain poorly understood.^3^, ^4^ Concerning the localization of the colon and CRC near the main visceral fat deposits (omental and mesenteric), it becomes clear that the TME is greatly populated by AT‐resident cells. Recent discoveries have shed light on this matter, indicating the importance of AT and obesity in the development and invasiveness of cancer.^2^, ^5^, ^6^, ^7^

White adipose tissue (WAT) is a heterogeneous organ whose role has been reduced to the storage of body fat for a considerable period. However, recent studies have revealed its function as a secretory structure responsible for the production of a prominent group of compounds, referred to as adipokines, along with cytokines, chemokines, and growth factors. They act paracrinely on adipose tissue cells and endocrinely, modulating the function of other tissues and organs.^2^, ^5^, ^8^ The production of such a great variety of molecules is a result of the presence of assorted cell types, including adipocytes, which represent the main percentage of WAT cells; the stromal vascular fraction (SVF), which comprises adipose stem (stromal) cells (ASCs); endothelial precursor cells; regulatory T cells (Tregs); macrophages; smooth muscle cells; pericytes; and preadipocytes. These biologically active effectors regulate physiological processes and contribute to the development of pathological states, including the promotion and progression of various cancers. This is particularly noticeable in obesity, in which the secretory balance of the WAT is dysregulated.^9^, ^10^

Obesity is defined as a disproportion between height and weight resulting from excessive fat accumulation and adipose tissue overgrowth and is usually accompanied by chronic, non‐acute systemic inflammation and insulin resistance.^3^ The typical histological picture of adipose tissue in individuals affected by obesity includes an increased number of adipocytes and their hypertrophy caused by the excessive accumulation of intracellular fats. There was also an increase in the number of adipose tissue‐infiltrating immunological cells, mainly macrophages and monocytes, attracted by factors released by abnormal and dead adipocytes.^11^, ^12^

Various types of adipose tissue cells (adipocytes, adipose stromal cells, and immunological cells) are elements that constitute the CRC microenvironment.^13^, ^14^ Their significant involvement in the regulation of metabolic processes, as well as their immunomodulatory capacities, make them important players in CRC progression, which is attributed among other things to the ability of WAT to produce compounds with broad biological activity.^15^, ^16^

Mutual interactions between adipocytes and CRC cells may lead to the upregulation of cancer cell migration, invasion, and proliferation rates as well as the transformation of adipocytes into cancer‐associated adipocytes (CAAs).^14^, ^17^, ^18^ These cells are characterized by an increased production of pro‐inflammatory molecules and are involved in metabolic processes related to the production of high‐energy compounds released into the extracellular matrix (ECM), which positions them as enhancers of carcinogenesis.^18^, ^19^

It should as well be taken into account that adipose tissue is one of the predominant sources of adult stem cells, which, according to recent reports, may be implicated in the formation and progression of gastrointestinal tumors.^20^, ^21^, ^22^ Through various direct and indirect interactions with CRC cells, ASCs may play a role in tumor niche formation, the development of cancer stem cells (CSCs), and the maintenance of CSC stemness. Notably, ASCs are relevant in the upregulation of CRC cell progression characteristics and epithelial‐to‐mesenchymal transition (EMT) acceleration through the secretion of factors such as hepatocyte growth factor (HGF) and interleukin 6 (IL‐6).^22^, ^23^, ^24^

In obesity, a relevant role is played by the coexisting low‐grade inflammation that participates in WAT‐associated tumorigenesis.^25^ The disrupted secretory profile of adipocytes shifted towards increased production of pro‐inflammatory cytokines, which, in common with metabolic dysregulation often accompanied by nutrient accumulation, provides to the recruitment of immunological cells and further alteration of the WAT secretory profile.^25^, ^26^ It is not well established whether nutrient accumulation may cause recruitment of immunological cells. More precise nutrient accumulation is associated with metabolic deregulation, which may take part in recruitment of immunological cells.

Concerning the complex interactions between CRC cells and adipose tissue cells, due to the multicellular composition of WAT, our focus was directed towards the role played by individual cell type (adipocytes, ASCs, and immunological cells) in CRC initiation and progression. Here, we concentrate on their contribution to TME formation and the processes related to CRC development and metastasis.

## 2 WHITE ADIPOSE TISSUE AS THE SOURCE OF DIVERSE TYPES OF CELLS

Adipocytes are the primary structural and functional components of WAT. However, as a complex secretory organ, this tissue exhibits much wider cellular diversity. In addition to adipose cells, WAT comprises adipose stem cells as well as immunological, nerve, and vascular cells. These integral structural components ensure high diversity in tissue functionality and contribute to the coordination of the physiological and pathological processes controlled by WAT.^13^, ^27^, ^28^

The major components of WAT are adipocytes surrounded by richly innervated and vascularized loose connective tissues.^11^ Typically, they are spherical cells, with a diameter ranging dramatically from about 20 μm to even 300 μm, depending on the triglycerides (TAGs) level. The main components of adult adipocytes are large lipid droplets that cover approximately 90% of the cell volume and force the peripheral position of other organelles.^28^, ^29^ These cells play crucial roles in fat storage and metabolism. The fatty acids required for the synthesis of TAG‐rich lipid droplets primarily originate from circulating lipoproteins. Upon their hydrolysis, free fatty acids (FFAs) are released and subsequently passively and actively transported to adipose cells via fatty acid transporters.^30^, ^31^ FFA can also arise from de novo lipogenesis (DNL) with glucose as the main substrate. Fat stored in adipocytes can be mobilized during periods of high energy demand through lipolytic processes. The classical lipolytic pathway requires the activity of three neutral lipases responsible for the hydrolysis of TAG to glycerol and fatty acids.^32^, ^33^ Several alternative pathways for this process have been described.^32^ However, their contribution to the lipolysis of TAG in lipid droplets and their participation in the development of pathological processes remain poorly understood. Adipocytes catabolize glucose through glycolysis, leading to the production of high‐energy molecules such as pyruvate, lactate, and possibly ketone bodies. These processes may contribute to the transition from white to beige adipocytes.^32^, ^34^ Beyond these metabolic functions, adipocytes modulate and regulate a wide variety of physiological processes through the secretion of biologically active molecules, acting not only as paracrine signaling agents but also by releasing them into the circulation in distant tissues and organs.^5^

ASCs are multipotent cells that can differentiate into multiple cell lineages derived from all three germ layers, the mesoderm, endoderm, and ectoderm, both in vitro and in vivo.^35^, ^36^ Although there are many terms for this cell fraction that can be found in the literature, we would like to use the abbreviation “ASCs” to refer to “adipose stem cells,” “adipose stromal cells,” and “adipose‐derived stem cells.” According to the International Society for Cellular Therapy (ISCT) and International Federation of Adipose Therapeutics and Science (IFATS), criteria for identifying cells as ASCs, apart from the ability to differentiate, include plastic‐adherence and specific surface molecule composition: the presence of CD105, CD73, and CD90, as well as the lack of CD45, CD34, CD14, CD11b, CD79α, CD19, and human leukocyte antigen–DR isotype (HLA‐DR).^36^, ^37^ ASCs may be isolated mechanically or enzymatically from the SVF, and their content may vary between 1% and 10%. The number of these cells may fluctuate depending on the WAT deposit used for their isolation, with a shift in efficiency towards the subcutaneous location.^36^, ^38^, ^39^ Most often, ASCs are localized perivascularly, probably because of their involvement in signaling connected to vasculogenesis, angiogenesis, and adipocyte development.^40^, ^41^ ASCs have the ability to secrete cytokines, adipokines, and other growth and angiogenic factors such as IL‐6, interleukin 7 (IL‐7), tumor necrosis factor α (TNFα), chemokine (C‐C motif) ligand 5 (CCL5), platelet‐derived growth factor (PDGF), HGF, granulocyte colony‐stimulating factor (G‐CSF), and macrophage colony‐stimulating factor. Therefore, they can interact with surrounding cells and tissues and may play a role in the emergence of various pathological states. ASCs undergo a two‐stage differentiation process that leads to the formation of mature adipocytes.^42^, ^43^, ^44^ First, the cells differentiate into preadipocytes, which are the immediate precursors of mature cells. Although this process does not involve morphological changes, the resulting cells lose their multipotent differentiation potential. The second stage, known as “terminal differentiation,” implies events connected with the acquisition of the morphological and functional characteristics of mature adipocytes.^45^, ^46^

Physiological images of adipose tissue are inevitably associated with the secretion of biologically active compounds such as adipokines. They have been implicated in the regulation of lipid and glucose metabolism, insulin sensitivity, inflammation, cardiovascular function, thermogenesis, and a broad spectrum of pathological processes. Most recognized among these are adiponectin, leptin resistin, visfatin, apelin, IL‐6, interleukin 8 (IL‐8), monocyte chemoattractant protein 1 (MCP‐1), TNFα, Plasminogen activator inhibitor‐1 (PAI‐1), and many others. The composition and amount of these may vary depending on the tissue location, size, cellular composition, and coexisting dysfunction.^2^, ^5^, ^6^

Immune cells are essential components of the WAT and are implicated in the maintenance of its functionality, especially in the development of pathological states. This fraction included macrophages, dendritic cells (DCs), innate lymphoid cells (ILCs), granulocytes (neutrophils, eosinophils, and basophils), lymphocytes (T and B), mast cells, and natural killer (NK) cells.^47^, ^48^ Among the aforementioned, macrophages are the most prevalent group, accounting for approximately 5% of the WAT cells.^47^, ^49^ Under physiological conditions, WAT‐inhabiting macrophages are characterized by a set of surface markers, CD206+, CD301+, and CD11c−, which classify them as M2 alternative activated cells, involved in immunosuppression processes.^50^, ^51^ They are involved in the removal of dead adipocytes through phagocytosis and may inhibit adipocyte precursor differentiation, thereby regulating the number of adipocytes in WAT.^50^, ^52^ Their polarization is mediated by interleukin 4 (IL‐4), which is mainly secreted by intratissue eosinophils in an interleukin 13 (IL‐13)‐dependent manner.^51^ Both these cytokines are also produced by adipocytes in WAT, suggesting their involvement in the polarization process.^53^ The role of innate DCs is still poorly understood, mostly because of the challenging nature of their separation from other immune cell fractions (mostly macrophages).^54^ They have been attributed to produce interleukin 10 (IL‐10), an anti‐inflammatory regulator of adipose tissue.^55^, ^56^ Another highly abundant fraction of WAT innate immune cells is type 2 ILCs (ILC2), which are important producers of interleukin 5 (IL‐5) and IL‐13. The factors they secrete affect the recruitment of eosinophils and therefore the accumulation of M2 macrophages.^57^, ^58^ Preservation of the M2 macrophage phenotype is also mediated by NKT cells as a result of IL‐4, IL‐10, and IL‐13 production.^59^, ^60^ In addition, they are responsible for interleukin 2 (IL‐2) secretion and facilitate the expansion of Tregs.^61^, ^62^ Among the cells engaged in adaptive immune processes, Tregs are one of the most important. Their anti‐inflammatory effect contributes to immune suppression and is therefore a mechanism for the reduction of insulin resistance development.^63^, ^64^ One of the potential pathways implicated in this process concerns Treg production of hydroxyprostaglandin dehydrogenase, which converts prostaglandin E2 (PGE2) into 15‐keto PGE.^65^, ^66^ WAT also contains a subset of regulatory B lymphocytes (Bregs), which, in addition to producing antibodies, mediate macrophage polarization towards the M2 phenotype. This type of immune cell can also produce IL‐10 and tumor growth factor β (TGFβ), thereby aiding in the modulation of the inflammatory response.^67^, ^68^ Another important group inhabiting WAT is mast cells, which are significantly localized to adipocytes. Owing to the secretion of a vast number of diverse biologically active compounds (cytokines, prostaglandins, and proteases), these cells affect ASC differentiation, adipocyte proliferation, and angiogenesis.^69^, ^70^, ^71^ Moreover, mast cells may regulate fat metabolism and contribute to the inflammation‐related dysregulation of obesity.^72^ Therefore, it is evident that immune cells are widely distributed in WAT, forming a complex network of interactions and contacts that ensure the preservation of WAT homeostasis.

## 3 OBESITY‐RELATED WHITE ADIPOSE TISSUE CHANGES, COEXISTING DYSFUNCTIONS

Metabolic imbalances caused by excessive dietary intake result in adipose tissue remodeling, which is primarily associated with changes in the number and morphology of mature adipocytes.^73^ Adipocyte hypertrophy is one of two pathways of adipose tissue enlargement associated with reduced metabolic plasticity and increased cellular stress. TAG accumulation causes a significant increase in the cell size, which can lead to IR after reaching a critical value.^74^, ^75^ Excessive lipid accumulation in the adipocytes can result in cellular overload and ectopic lipid deposition.^76^, ^77^ WAT expansion via the hyperplastic pathway requires the recruitment of new ASCs from the SVF and their differentiation into mature adipose cells. Both recruitment and differentiation are driven by paracrine factors secreted by hypertrophied mature adipocytes.^74^, ^78^ In vivo studies using mouse models have indicated that adipocyte hypertrophy occurs much earlier than tissue hyperplasia.^79^ Experiments with stable isotope labeling experiments identified hypertrophy as the main pathway for adipose tissue outgrowth.^80^, ^81^ Therefore, expansion through the recruitment of new cells is thought to be a compensatory mechanism in a state of overnutrition in an attempt to remedy the development of metabolic disruptions.^77^

Obesity is characterized by an abnormal fat metabolism caused by the excessive release of FFA from hypertrophic adipocytes.^82^, ^83^ Under these conditions, basal (spontaneous) lipolytic activity in adipocytes increases. Changes in lipolysis are associated with the leakage of unesterified FFAs from massively enlarged cells, which may be related to fluctuations in perilipin (PLIN) levels, which protect lipid droplets from lipase action.^84^, ^85^, ^86^ Moreover, emerging insulin insensitivity implies a significant elevation of lipolysis in hypertrophic adipocytes, whereas smaller insulin‐sensitive cells may present a higher lipogenesis/lipolysis ratio.^87^, ^88^ Multiple studies have indicated that a WAT cholesterol imbalance is associated with obesity state. Enlarged adipocytes accumulate cholesterol in lipid droplets. However, insulin‐resistant overloaded cells could possibly release stored cholesterol, leading to its deposition in other organs.^89^, ^90^ The development of obesity is accompanied by alterations in the levels of factors produced by the adipose tissue. These include the substances secreted by abnormal adipocytes, ASCs, and accumulated immune cells.^91^ Several adipokines are associated with adipose‐tissue enlargement. Among more than 600 potential adipokines identified to date, those with the best‐studied roles in obesity are leptin, adiponectin, visfatin, resistin, chemerin, and apelin. In the obese state, the levels of most of these biomarkers increased, except for adiponectin, which was downregulated.^89^, ^92^, ^93^ Cytokines play a prominent role in the development of obesity and concomitant disorders. One of the most recognized among them is TNFα, whose level positively correlates with the obesity progression and the development of insulin resistance in peripheral tissues.^94^, ^95^ TNFα induces the release of fatty acids from TAG, affecting the action of PLINs.^96^, ^97^ Furthermore, it has been implicated in the modulation of the insulin signaling pathway and the regulation of insulin secretion.^97^, ^98^ In mouse models, anti‐TNFα therapy has been shown to improve insulin sensitivity and also lower plasma fatty acid levels.^98^, ^99^ Another crucial pro‐inflammatory factor, IL‐6, has been found at increased concentrations in individuals affected by obesity. Overexpression of this cytokine is mostly attributable to the accumulation of immune cells; however, adipocytes themselves contribute to total IL‐6 levels, mainly before immune cell recruitment.^100^, ^101^ Moreover, the upregulation of lipolysis, fat oxidation, and insulin resistance has been reported in individuals with elevated plasma IL‐6 levels.^102^, ^103^ Interleukin 1β (IL‐1β) is secreted by WAT, and its higher levels have been found in patients with obesity.^104^ This may be a direct cause of insulin resistance through the induction of pancreatic inflammation, leading to apoptosis and the onset of this disorder.^105^, ^106^ In addition, it is suggested that hypertrophied adipocytes may accelerate the infiltration of adipose tissue by macrophages, mainly through excessive secretion of MCP‐1. Macrophages themselves are also able to secrete MCP‐1, leading to the further recruitment of immune cells and the development of inflammation. This effect is preferentially observed in visceral adipose tissue.^107^, ^108^

Changes in the number of adipocytes in obese mice may lead to apoptosis or secondary necrosis. Their deaths, together with FFA release and production of the aforementioned pro‐inflammatory factors (IL‐6, TNFα, and MCP‐1), enhance recruitment and activation of immune cells and thus the development of inflammation. This is associated with changes in the immunological profile of WAT, including pro‐inflammatory cells such as macrophages, mast cells, neutrophils, DCs, CD8+ T cells, Th1 cells, and B cells.^109^, ^110^, ^111^ Crown‐like structures formed by the macrophage encirclement of dead adipocytes attracted by MCP‐1 are typical of adipose tissues affected by obesity.^112^, ^113^ The predominant populations of innate WAT macrophages in patients with obesity are CD9+ cells residing in crown‐like structures and Ly6C+ cells located outside them. CD9+ cells are lipid‐laden cells involved in triggering pro‐inflammatory responses.^112^, ^114^

Critical to the development and maintenance of inflammation in adipose tissue are classically activated M1 macrophages, which represent the largest percentage of immune cells in the tissues of individuals with obesity.^115^, ^116^ Metabolic changes in the WAT of individuals with obesity lead to the activation of the Rho‐associated kinase/c‐Jun N‐terminal kinase (ROCK/JNK) and ROCK/extracellular signal‐regulated kinase (ERK) pathways, which result in monocyte and macrophage polarization towards the M1 type.^117^, ^118^ In the early stages of obesity, the number of pro‐inflammatory ILCs group 1 (ILC1) increases.^119^, ^120^ Their ability to produce interferon‐gamma (IFNγ) promotes macrophage M1 polarization and adipose tissue fibrogenesis.^121^, ^122^ The number of ILCs in Group 2 (ILC‐2) was, in turn, reduced, which was accompanied by a decrease in the number of eosinophils and M2‐like macrophages.^58^, ^123^ Neutrophils are another group of immune cells that accumulate in individuals with obesity within the first few days of starting a fat‐rich diet.^124^, ^125^ Their recruitment may be mediated by activated macrophages via the secretion of nucleotide attractants. Neutrophils are characterized by increased production of myeloperoxidase and IL‐8 as well as upregulated matrix metalloproteinase 9 (MMP9) expression.^48^, ^126^ The similarity in the surface marker composition of DCs and macrophages makes them difficult to distinguish. Thus, for years, reports on the role of DC in obesity are limited. Their pro‐inflammatory activity is mediated by elastase, the levels of which increase in the obese state. Recent studies indicated that DCs can be distinguished from macrophages based on a specific subset of surface markers, making research on their contribution to WAT inflammation and insulin resistance possible.^127^, ^128^, ^129^ Their role is most likely based on their ability to present antigens and activate other immune cells (e.g., CD4+ T cells).^128^, ^130^ Alterations in immune cell content within the WAT of individuals with obesity also affect T cell populations. The numbers of CD8 + Th1 and Th17 cells in WAT tend to increase significantly with the development of obesity, whereas the percentages of Th2 and Treg cells decrease.^131^ Based on IFNγ content in WAT of individuals with obesity, it is postulated that CD4+ T cells undergo polarization towards Th1 lymphocytes, what contributes to metabolic dysfunction and pro‐inflammatory reprogramming.^131^, ^132^ CD8+ T cells mediate inflammation in WAT, promote macrophage recruitment, and mitigate ILC2 and eosinophil populations in WAT.^133^, ^134^ The IL‐6‐dependent accumulation of Th17 lymphocytes in WAT may also support the propagation of obesity‐associated pro‐inflammatory responses.^127^, ^135^ Tregs and Th2 anti‐inflammatory cells have protective properties, limiting inflammation and the development of type 2 diabetes in individuals with obesity.^131^, ^136^

Obesity is associated with alterations in the penetrating vasculature. Many past studies have indicated the presence of capillary rarefaction as a consequence of metabolic dysfunction and adipose tissue outgrowth.^137^, ^138^ Metabolic and immunological deregulation within the enlarging WAT, together with adipocyte death caused by hypoxia of the overgrowing tissue, modulate the de novo formation of capillaries. Hypoxia activates the hypoxia‐inducible factor (HIF) signaling pathway within intrinsic macrophages, resulting in increased production of PDGF and upregulation of inflammatory processes.^139^, ^140^, ^141^ Several previous studies have highlighted the important role of the vascular endothelial growth factor (VEGF) in vascular growth in obesity. Elevated levels of this factor promote angiogenesis, and its deficiency in the WAT may result in increased metabolic dysfunction.^142^, ^143^, ^144^

Obesity‐associated adipose tissue remodeling is a complex network of interactions between different cell types residing in WAT arising from functional reprogramming and additional recruitment. It also alters the shape of the secretory profile of tissues affected by obesity, resulting from the interaction between obese adipocytes, SVF cells, and accumulated immune cells. Recent studies have shed light on the role of immune and stem cells in the pathogenesis of obesity‐associated inflammation and metabolic dysregulation, although the complexity of the existing interactions necessitates additional research in order to complete our understanding.^5^, ^20^, ^74^, ^91^, ^92^

## 4 ROLE OF ADIPOCYTES IN COLORECTAL CANCER PROGRESSION

As a secretory organ, the WAT modulates cancer progression through the production of adipokines. Adiponectin, resistin, ghrelin, and Nicotinamide Phosphoribosyltransferase (NAMPT) CCAAT/enhancer‐binding protein‐alpha are the most studied factors in terms of their impact on CRC progression.^13^, ^15^ A plethora of articles have investigated the involvement of the aforementioned compounds, as well as other WAT‐secreted molecules, in tumor promotion. However, many WAT‐releasing factors remain unknown or are poorly described. The effects of these compounds on CRC cells of those already characterized are summarized in Tables 1 (adipokines) and S1 (cytokines).

**TABLE 1:** Secretory profile of adipokines and other factors released by cells typically inhabiting adipose tissue on colorectal cancer progression and fluctuations in their level under obesity condition.

Molecule	Expression by AT cells	Importance in CRC		Level under obesity
		In vitro	In vivo	
Adiponectin	Adipocytes of WAT^145^	Reduction of CRC cell growth, viability, migration,^146^ colony formation, adhesion and invasion^5^, ^91^, ^147^	Antitumor activity during the early stages of CRC^148^	↓ in serum ↓ mRNA in SAT of morbidity women with obesity^149^
Resistin	Preadipocytes, adipocytes, peripheral blood mononuclear cells^150^; WAT‐derived macrophages^151^; Monocytes^10^	Enhancement of metastasis^152^; Decline the proliferation rate^153^	Positive correlation between serum level and the proinflammatory state of colorectal cancer^154^	↑ in serum^155^ ↑ mRNA in VAT of morbidity women with obesity^149^
Visfatin (NAMPT)	Adipocytes^156^; WAT‐infiltrating macrophages^151^	Promotion of migration and invasion of CRC cells^157^; Protection the CRC cells from oxidative stress induced by inflammation^158^; Decrease colon cancer cell apoptosis and promoting proliferation^159^	Upregulated in adenoma and adenocarcinoma tissues from CRC patients^158^; Negative correlation with prognosis of CRC patients^157^	↑ in plasma^160^
Apelin	Adipocytes^161^	Stimulation the proliferation,^162^ the migration and invasion of colon cancer cells^161^, ^162^		↓ in plasma of young people^163^; ↑ in plasma^164^
IGF	IGF‐I Adipocytes, stromal vascular fraction cells^165^	IGF‐I Role of IGF‐I receptor in the stimulation of CRC cells proliferation and inhibition of apoptosis^166^	IGF‐I Association between higher IGF‐I concentrations and colorectal cancer risk^167^; Important role in colorectal cancer initiation, development, progression and metastasis^168^	IGF‐I ↑circulating levels^169^; ↓ total and free levels in patients with obesity^170^
	IGF‐II Unidentified cells of AT^74^, ^171^	IGF‐II Stimulation the proliferation and cell–cell/cell–ECM contact^172^	IGF‐II Positive correlation of high levels of IGF‐II with risk of developing, progression and relapse of CRC patients^172^	IGF‐II ↑ circulating levels^169^
IGFBPs	IGFBP‐2, ‐4,‐ 6 Adipocytes^171^ IGFBP‐3 Preadipocytes, adipocytes^171^; Adipocytes stromal vascular fraction cells^165^ IGFBP‐5 Unidentified cells of AT^74^, ^171^ IGFBP‐7 Unidentified cells of AT^173^	IGFBP‐2 Promotion the proliferation and migration of CRC cells^174^ IGFBP‐3 Induction of proliferation of CRC cells^175^; IGFBP‐6 Promotion of migration of colon cancer cells^176^	IGFBP‐3 Correlation between lower expression and better survival outcome in CRC patients^177^; The elevation of IGF‐1/IGFBP‐3 ratio and the reduction of IGFBP‐3 may be related to the initiation of CRC^178^; Reduction of tumor growth in colorectal cancer xenograft model^179^; IGFBP‐5 Plausible pro‐cancerous effect^180^	IGFBP‐2 ↑circulating mRNA level^181^ IGFBP‐3 ↑ plasma level after an overnight fast before overeating^182^ IGFBP‐4 ↓ mRNA level in blood ^181^ IGFBP‐5 ↓ mRNA level in blood ^181^ ↑ mRNA level in the omental AT in comparison to SAT IGFBP‐7 ↑circulating mRNA concentration^181^
VEGF	TAMs, macrophages M2^183^; Polymorphonuclear leukocytes, monocytes^184^	Enhancement of proliferation, migration, and invasion of CRC cell lines^185^	Support for tumor angiogenesis and growth^186^	↑ serum concentration of VEGF‐A, ‐B, ‐C, ‐D and soluble VEGF receptor‐2^187^, ^188^; ↓ blood level of VEGF‐D^187^

Abbreviations: AT, adipose tissue; CRC, colorectal cancer; ECM, extracellular matrix; IGF, insulin‐like growth factor; IGFBP, insulin‐like growth factor binding protein; PBMCs, peripheral blood mononuclear cells; SAT, subcutaneous adipose tissue; TAM, tumor‐associated macrophages; VAT, visceral adipose tissue; VEGF, vascular endothelial growth factor; WAT, white adipose tissue; ↑, higher level; ↓, lower level; ‐, no significant differences.

Owing to the physical contact of the colon with visceral fat deposits in the human body, the molecular crosstalk between WAT and CRC may be even more direct.^189^ Thus, adipocytes and other WAT cells are involved in the formation of the TME at different stages of CRC progression. Adipocytes exhibit a high degree of plasticity in response to changes in the surrounding microenvironment, and the modifications they undergo can be observed under conditions of overnutrition, skin fibrosis, or dedifferentiation during lactation.^190^, ^191^ Given this potential, it seems understandable that as elements of the CRC microenvironment, adipocytes are reprogrammed through interactions with cancer cells. This transdifferentiation process results in the formation of smaller cells with a fibroblast‐like phenotype (CAAs) that lack specific markers for differentiated adipocytes and have reduced intracellular lipid content.^18^ Repression of CCAAT/enhancer‐binding protein‐alpha (C/EBPα) and Peroxisome proliferator‐activated receptor gamma (PPARγ) Inducible nitric oxide synthase expression, which is mediated by factors such as TGFβ and TNFα secreted by CRC cells, appears to be one of the potential mechanisms implicated in the process of adipocyte dedifferentiation. Moreover, TNFα may affect adipocyte lipolysis by increasing Inducible nitric oxide synthase iNOS levels and downregulating PLIN expression, leading to increased hormone‐sensitive lipase activity and CAA formation. Factors, such as matrix metalloproteinase 11 (MMP11), PAI‐1, IL‐6, IL‐8, Wnt Family Member 3A (Wnt3a), and Wnt Family Member 5A (Wnt5a), are thought to be involved in this process.^192^, ^193^, ^194^

**FIGURE 1:**
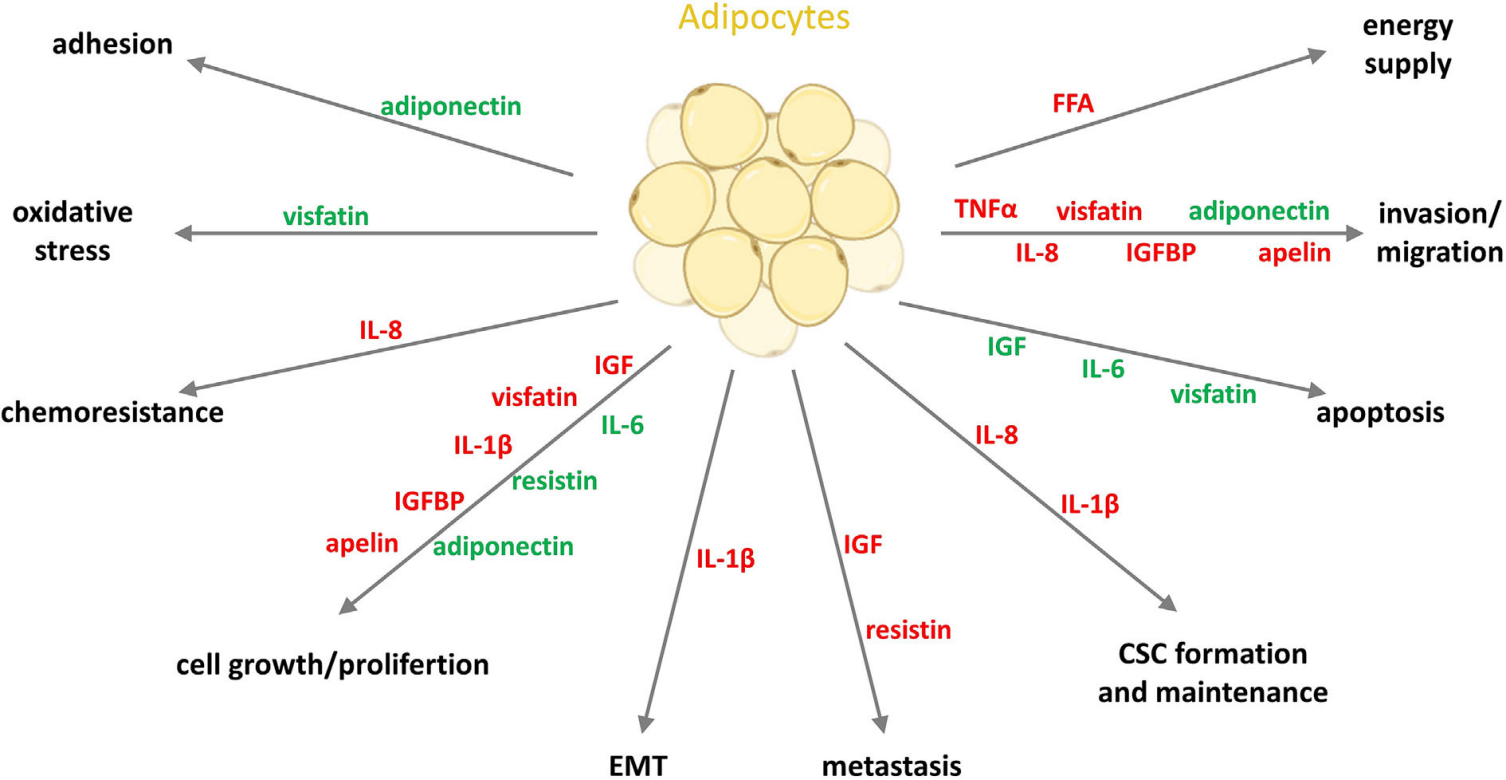
The impact of molecules released by adipocytes on colorectal cancer cells. The stimulating effect is marked in red, and the inhibitory effect on colorectal cancer (CRC) progression is marked in green (created with BioRender). CSC, cancer stem cell; EMT, epithelial–mesenchymal transition; FFA, free fatty acid; IGF, insulin‐like growth factor; IGFBP, insulin‐like growth factor binding protein; IL‐1β, interleukin‐1β; IL‐6, interleukin‐6; IL‐8, interleukin‐8; TNFα, tumor necrosis factor α. (created with BioRender).

CAAs are an integral part of the TME and contribute to enhanced CRC progression through direct and indirect interactions with cancer cells (Figure 1). Treatment with adipocyte‐conditioned medium increases the proliferation, migration, and invasion of CRC cells, highlighting the contribution of adipocyte‐secreting molecules.^2^, ^195^, ^196^ This effect may be associated with a disturbed secretion profile of CAAs, shifted towards increased production of pro‐tumorigenic factors such as CCL5, chemokine (C‐C motif) ligand 2 (CCL2), and C‐X‐C motif chemokine ligand 8 (CXCL8), IL‐6, IL‐1β, TNFα, VEGF, and leptin.^192^, ^197^, ^198^ Moreover, overgrowth of adipose tissue in individuals with obesity can increase the number of adipocytes responsible for WAT secretory ability and affect the content and levels of the produced factors. CRC patients with obesity show elevated levels of pro‐inflammatory and pro‐angiogenic molecules compared with lean and non‐cancer patients, suggesting a contribution of WAT and innate adipocytes to CRC initiation and development.^1^, ^199^ This, in turn, may be associated with a deterioration in the condition of patients with CRC and, bearing in mind the role of proangiogenic factors, an increased potential for metastasis.

The ECM is a key component of the TME, and it has been shown to be involved in the regulation of tumor growth and invasive capacity. Adipocytes within the TME may participate in desmoplastic processes because of the upregulated expression of proteins associated with ECM remodeling. These include MMP11, PAI‐1, procollagen‐lysine,2‐oxoglutarate 5‐dioxygenase 2 (PLOD2), as well as the predominant structural component of the ECM in WAT, collagen type I.^192^, ^193^, ^200^ Alterations in the levels of ECM remodeling factors, particularly in obesity, lead to enhanced crosslinking of collagen fibers, excessive ECM deposition, and fibrosis. These obesity‐associated modifications promote tumor progression at different stages of development, stimulate tumor cell growth, and contribute to the malignant transformation of human epithelial cells.^201^, ^202^, ^203^ Furthermore, matrix metalloproteinase 14 (MMP14), which is highly expressed in the WAT of individuals with obesity, may play an important role in remodeling processes and established role in cancer development. However, the mechanisms underlying the effects of this enzyme on CRC progression remain unclear.^204^, ^205^

CAAs recruited within the CRC microenvironment show a predominance of catabolic metabolism, resulting in the release of high‐energy compounds such as lactate, pyruvate, and ketone bodies into the environment.^17^, ^189^ Fast‐proliferating and rapidly growing cancer cells require more energy than normal tissues. Thus, the presence of high‐energy molecules in their surroundings enhances their progression and metastatic properties.^189^ CAAs provide energy to cancer cells by releasing endogenous fatty acids from degraded intracellular lipid droplets. The breakdown of lipid droplets can be further accelerated by neighboring tumor cells, which capture subsequent FFAs and direct them into the fatty acid β‐oxidation (FAO) pathway. These lipolytic impairments within CAAs may result in the metabolic reprogramming of cancer cells.^189^, ^206^, ^207^ The mechanism of FFA uptake by tumor cells has not been uniformly defined; however, it is known that this process may be mediated by specialized transporters such as cluster of differentiation 36 (CD36), fatty acid‐binding proteins (FABPs), and the fatty acid transport protein family (FATPs). Part of this interplay has been observed in CRC, melanoma, and ovarian, prostate, and breast cancers, suggesting that it might be considered a typical link between the adipocyte‐rich TME, obesity, and cancer.^206^, ^208^, ^209^, ^210^ In addition, FAO activation in cancer cells is thought to be a pro‐tumorigenic mechanism associated with stemness maintenance and cell proliferation.^207^ Moreover, CRC cells modify the release of fatty acids from adipocytes by promotion of adipocyte browning. To date, it has been shown that this effect may involve exosomal miR‐146b‐5p translocation from CRC cells and downregulation of migration and invasion inhibitory protein (MIIP) expression, leading to the elevation of N‐linked alpha‐2‐glycoprotein 1 and zinc‐binding (AZGP1) glycosylation via the cyclic adenosine monophosphate ‐Dependent Protein Kinase (cAMP‐PKA) pathway.^211^, ^212^ Accumulated FFA within CRC cells may constitute a source of lipid droplet formation, which is a key factor in cell proliferation and development.^213^, ^214^ This mechanism represents a potential therapeutic target for new anti‐cancer therapies that triggers cancer cell metabolism and growth.

Adipocytes within the TME are able to increase the expression of carnitine palmitoyltransferase 1A (CPT1A), a key rate‐limiting enzyme in FAO, through a PPARγ‐dependent pathway. It has been reported that this modification can accelerate tumor promotion and initiation and may be an important element in the maintenance of cancer cell stemness.^215^, ^216^, ^217^ Additionally, CPT1A may facilitate the tolerance of cancer cells to hypoxic environments by enhancing mitochondrial fatty acid oxidation. Moreover, it was found that the CPT1A complex accelerated cell proliferation through an FAO‐independent pathway.^215^, ^218^ Recent studies have suggested that CPT1A is a critical link in the communication between the adipocyte‐rich TME and cancer cells.^215^, ^217^

## 5 ADIPOSE‐DERIVED STEM CELLS AS A SIGNIFICANT EFFECTOR IN COLORECTAL CANCER AGGRESSIVENESS

Nonetheless, adipocytes are not the only cells within WAT capable of interacting with cancer cells. In this regard, there is increasing interest in cells derived from the SVF, especially adipocytes and ASCs. Recent studies have shown that the presence of ASCs increases CRC cell sphere formation, suggesting their involvement in tumor development. In addition to their tumor‐initiating abilities, ASCs have been shown to be able to enhance the growth rate of cancer cells, demonstrating their cancer‐accelerating properties. Moreover, promotion of proliferation was observed with the use of conditioned media collected from ASCs, suggesting the involvement of molecules produced and secreted into the environment during this process.^219^, ^220^ The observed effect may be mediated by IL‐6, whose production contributes to ASCs pro‐tumor properties. This effect is not unique to CRC, as analogous interactions have been noted in breast cancer.^22^ Another mode of interplay between ASCs and CRC cells is related to the activation of the ERK1/2 pathway, which is an important element in the regulation of the growth and survival of cancer cells. It has been postulated that this effect may be mediated by ASC‐driven galectin 3, which is considered a linking element in the mutual impact of ASC–CRC cells. The aforementioned alterations, attributed to the effects of galectin 3, were preferentially observed in senescent rather than premature ASCs.^221^

Another aspect relevant to tumor aggressiveness modulated by ASCs is the ability of tumor cells to undergo EMT. This is an evolutionarily preserved developmental process that is activated during cancer progression and contributes to metastatic properties.^222^ ASCs have been confirmed to be present in both primary and metastatic CRC foci of patients affected by obesity. These cells, by releasing biologically active compounds (HGF and IL‐6), promote EMT through the activation of signal transducer and activator of transcription 3 (STAT3) and zinc finger E‐box binding homeobox 2 (ZEB2) and contribute to the enlargement of the metastasis‐capable CD44v6+ cell population.^24^, ^223^ The increasing number of CD44v6+ cells is followed by the recruitment of new ASCs due to the production of factors such as neurotrophin‐3 (NFT3) and nerve growth factor (NGF), creating a feedback loop that amplifies the aggressiveness of CRC cells.^24^, ^224^ Furthermore, the CRC potential to undergo EMT is also possibly regulated by the expression of factors such as fibroblast growth factor 10 (FGF10), VEGF ‐ C, IL‐10, and TNFα. Their enhanced production by ASCs was observed upon co‐culture with CRC cells, which concomitantly exhibited upregulated expression of EMT‐related genes. A possible self‐perpetuating effect was also observed in that study, as FGF10 is probably the main factor related to the activation of ASCs, which then stimulates the EMT of cancer cells.^223^, ^225^ Additionally, it has been postulated that the EMT‐accelerating effect may be further intensified by direct or indirect ASC–CRC cell interactions in relation to the use of ASC‐conditioned medium. Some studies have indicated that tight physical contact is essential for the development of the mesenchymal phenotype in tumor cells.^226^

An indispensable element associated with the EMT mechanism is the maintenance of stemness among CSCs. This process is thought to be regulated by the interplay between tumor cells and ASCs. Factors secreted by ASCs that potentially play relevant roles in these interactions include IL‐8, HGF, and Jagged‐1.^23^, ^24^, ^227^ Recent research has demonstrated that the inhibition of IL‐8‐related signaling results in a decrease in the stemness, self‐renewal capacity, and EMT potential of CSCs.^23^ The presence of HGF has been correlated with the expression of genes (*CXCR4*, *SLUG*, *TWIST*, *ZEB1*, *ZEB2*) associated with CRC cell stemness and maintenance of CSCs.^24^ Furthermore, CRC expression of the surface molecule prominin‐1 (CD133), one of the best‐known markers of CSCs, was associated with EMT capacity and enhanced expression of N‐cadherin and vimentin.^23^, ^228^ Such a correlation may explain the intensified invasive capacity of CRC cells and suggest a link between the ability of ASCs to promote EMT and their stimulatory role in the formation of CSCs.

Recent studies have revealed the involvement of cancer‐associated fibroblasts (CAFs) as a cell involved in tumorigenesis and suggested their impact on the clinical picture of tumor development. However, their cellular origins remain poorly understood, making them challenging therapeutic targets.^229^ Coculturing ASCs with cancer cells, analogous to differentiated adipocytes, can trigger reprogramming pathways, resulting in the transformation of ASCs into CAAs and CAFs, both of which are typical of the TME.^228^, ^230^ This differentiation process has been identified in various types of cancers such as breast, colorectal, and pancreatic ductal adenocarcinomas.^230^, ^231^, ^232^ Its mechanism remains largely unexplored, which is further emphasized by the fact that CAF formation can also be observed when ASCs are co‐cultured with CSCs or cultured in the presence of exosomes isolated from CRC cells. CAFs formation is associated with elevated *TRPC3* expression, which may accelerate tumor progression in vitro.^23^, ^230^ The contribution of ASCs alone to the formation of the CAFs population may differ significantly in individuals with obesity, whose WAT volumes exceed physiological values. It has been demonstrated that ASCs isolated from individuals with a body mass index (BMI) above 30 kg/m^2^ had increased expression of markers for CAFs compared with cells isolated from patients with a BMI below 25 kg/m^2^.^20^, ^233^ Given the involvement of CAFs in cancer progression, their formation from ASCs provides further insight on the engagement of ASCs in tumorigenesis and allows for the formulation of new mechanisms linking tumor development with obesity.

ASCs, in addition to producing tumor‐supporting factors, may provide scaffolds for the growth of CRC cells. In direct coculture, the CRC cells preferentially attached to ASCs adsorbed on the surface instead of on the plastic bottom of the culture vessel. Moreover, the ratio of interacting cells shifted towards CRC cells, multiples of which simultaneously interacted with one ASCs.^228^ Research to date indicates that ASCs may function as a supportive layer, providing adhesion molecules and ECM components for CRC cells and enhancing their adhesion and engraftment capacity.^228^, ^234^ When considering the potential of ASCs to produce EMT‐modifying compounds and their beneficial effects on spheroid formation, their ability to directly interact with tumor cells could be of particular importance in metastatic processes.

**FIGURE 2:**
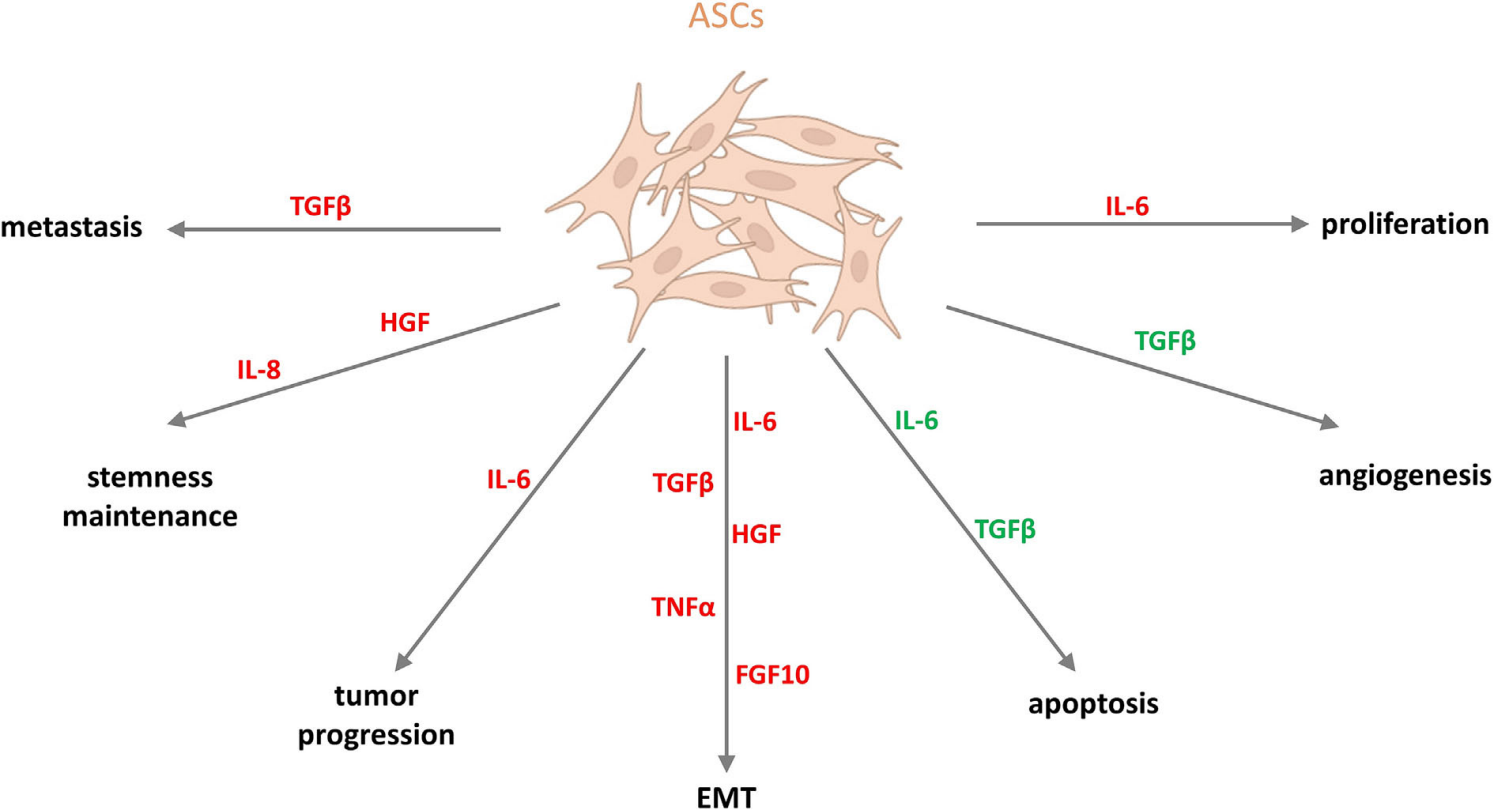
The influence of adipocyte stem cells on colorectal cancer (CRC) progression. The stimulation effect is marked in red, and the inhibitory effect on CRC progression is marked in green (created with BioRender). ASCs, adipocyte stem cells; EMT, epithelial–mesenchymal transition; FGF10, fibroblast growth factor 10; HGF, hepatocyte growth factor; IL‐6, interleukin‐6; IL‐8, interleukin‐8; TGFβ, tumor growth factor β; TNFα, tumor necrosis factor α. (created with BioRender).

Notably, the coculture of ASCs with CRC cells may contribute to lower intracellular reactive oxygen species (ROS) levels in ASCs.^228^, ^235^ This may provide another aspect of the interplay between ASCs and CRC cells and suggest a possible effect of CRCs on the increased survival of ASCs as a tumor‐promoting component. ASCs are rich sources of factors involved in tumorigenesis. They secrete biologically active compounds that partially overlap with those produced by other WAT cells; contribute to the increased proliferation, migration, and invasion of tumor cells; and may promote processes such as EMT, tumor formation, stemness maintenance, and angiogenesis.^20^, ^24^, ^224^, ^225^, ^228^ More details on the wide range of compounds secreted by ASCs are presented in Tables 1 and S1 as well as summarized in Figure 2.

## 6 WHITE ADIPOSE TISSUE‐RESIDENT IMMUNE CELLS AFFECT COLORECTAL CANCER PROGRESSION

To assess the impact of WAT resident cells on tumor progression, it is essential to consider the role of immune cells as an integral part of this functional subunit. Their percentage in the population of WAT‐building cells is significant, especially in the obese state, which, together with obesity‐related immune reprogramming, makes them crucial elements linking pathological adipose tissue overgrowth and tumor progression.^16^, ^236^

**FIGURE 3:**
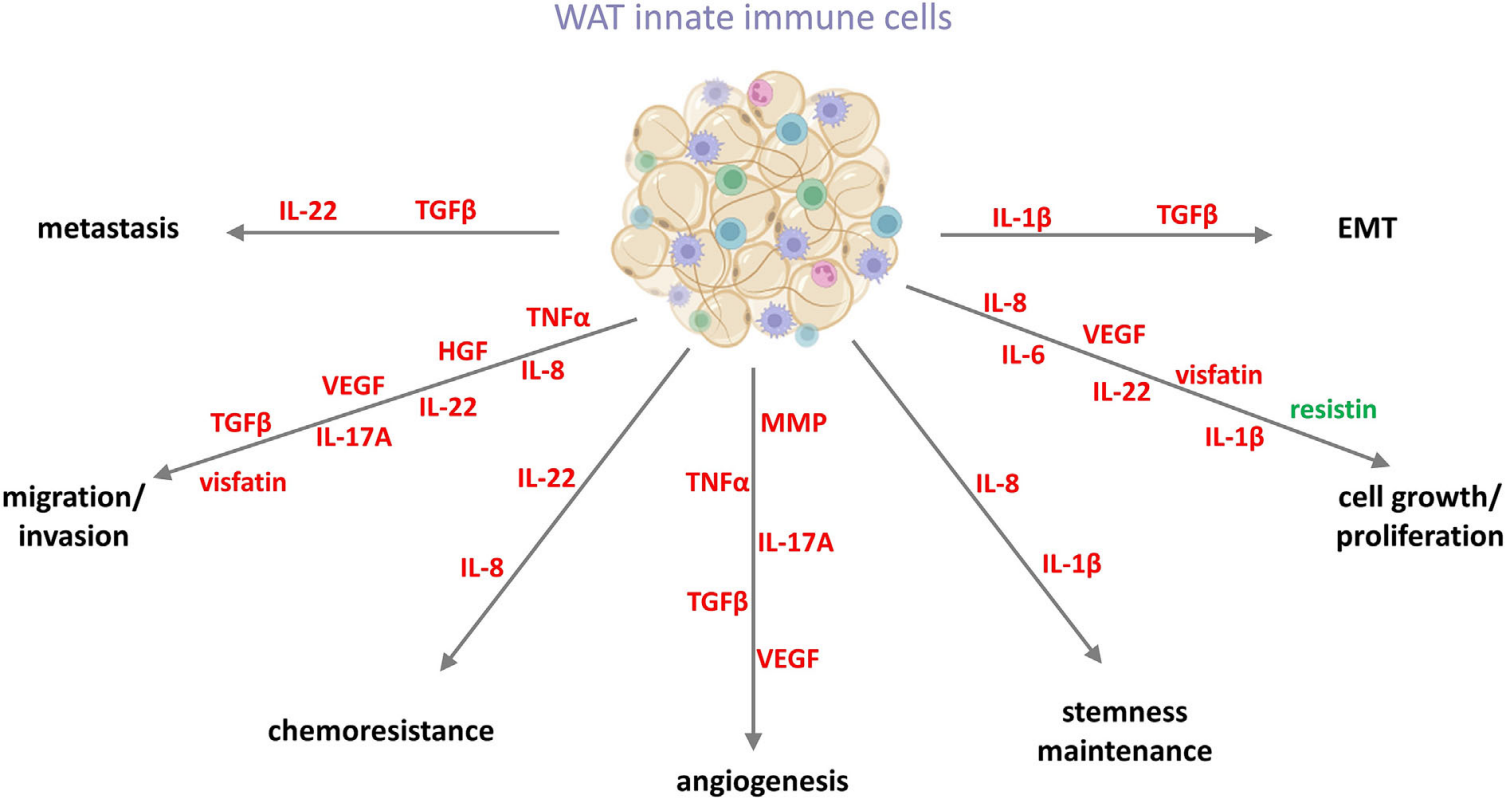
The effect of molecules released by immune cells resident in white adipose tissue (WAT) on colorectal cancer (CRC). The stimulating effect on CRC progression is marked in red and the inhibitory in green (created with BioRender). EMT, epithelial–mesenchymal transition; HGF, hepatocyte growth factor; IL‐17A, interleukin‐17A; IL‐1β, interleukin‐1β; IL‐22, interleukin‐22; IL‐6, interleukin‐6; IL‐8, interleukin‐8; MMP, matrix metalloproteinase; TGFβ, tumor growth factor β; TNFα, tumor necrosis factor α; VEGF, vascular endothelial growth factor. (created with BioRender).

Adipose tissue is a source of numerous factors involved in immunomodulatory processes. This capacity is heavily based on the presence of a diverse population of innate immune cells with rich excretory profiles, accompanied by adipocytes and ASCs that share this function.^5^, ^20^ Immune cells are characterized by highly diverse secretory abilities that are significantly altered in individuals with obesity. Numerous past studies have demonstrated that both pro‐ and anti‐inflammatory molecules may contribute to cancer progression^5^, ^237^ (Figure 3. The characteristics of the numerous factors produced by immunological cells residing in WAT are summarized in Tables 1 (adipokines) and S1 (cytokines).

The most abundant type of immune cells inhabiting WAT are macrophages, which are particularly important in the linkage between obesity‐related inflammation and cancer progression.^238^ The contribution of macrophages to the tumorigenic process is wide‐ranging, playing a role in either classically (M1) or alternatively activated (M2) cells. However, the high plasticity of macrophages in response to environmental changes results in the development of multiple functional phenotypes. This, combined with the ability to switch between M1 and M2 populations in the tumor environment and the existence of types with features of both or none of these types, makes the unambiguous classification of tumor‐associated macrophages (TAMs) challenging.^239^, ^240^ M1‐type macrophages participate mainly in the first stages of tumor development, producing reactive nitrogen and oxygen species that promote oncogene activation.^241^, ^242^ In further stages, they may enhance the formation of a pro‐inflammatory microenvironment, that at first may promote tumor development but mainly contributes to anti‐tumor immunity, which results in the production of chemokines typical for cells with a pro‐inflammatory profile, for example, IL‐1β, IL‐6, TNFα, and MCP‐1.^243^, ^244^ Moreover, M1 macrophages can promote the cytotoxicity of other leukocytes as a result of their increased ability to present tumor antigens, leading to the apoptosis of cancer cells. Alternatively, cells displaying typical pro‐cancer activities are activated by M2 macrophages. Co‐culture of macrophages with CRC cells increases the abundance of M2‐type cells, and current studies indicate the involvement of the phosphatidylinositol 3' ‐kinase/Protein kinase B/mammalian target of rapamycin kinase (PI3K/AKT/mTOR) signaling pathway in this process via Epidermal Growth Factor (EGF) secretion by CRC cells.^238^, ^245^ M2 macrophages recruited to the TME perform an important role in tumor progression, through the production of compounds enhancing and modulating angiogenesis (VEGF, MMP, TNFα, IL‐8), cancer cell migration (HGF, TGFβ, PDGF), and invasiveness (VEGF, EGF).^246^, ^247^ They appear to be associated with the development of CRC in patients with chronic gastrointestinal diseases, such as ulcerative colitis.^248^ In addition, it is worth pointing, that cancer‐related metabolic reprogramming of macrophages could be associated with their cancer‐promoting abilities. The M2 macrophages secrete ornithine, a product of arginine metabolism.^249^ Ornithine may promote tumor growth and metastasis by activation of interleukin 33 expression in cancer cells. Moreover, TAMs can promote CRC growth by inhibiting spermidine production through the increased expression of Lysophosphatidic Acid Acyltransferase (ABHD5).^243^, ^250^ Macrophages can interact with CAFs and CAAs within the TME and are mutually regulated.^251^, ^252^ Moreover, M2 macrophages may enzymatically reorganize the ECM in the surrounding tumor, which, given their interaction with CAFs, could serve as a significant mechanism implicated in tumor angiogenesis and enhanced invasiveness of cancer cells.^253^, ^254^ When taken together, these findings highlight additional implications between macrophages and WAT‐derived cells in the context of cancer progression and complicate a straightforward understanding of their interrelationships in the CRC microenvironment.

WAT is rich in diverse populations of immune cells that form a network of complex interactions capable of modulating tumorigenesis. They participate in both the enhancement of tumor growth and invasiveness or in the formation of anti‐tumor immunity, depending on the secretory profile of the particular cell subtype.^236^, ^255^ Changes in the contents of individual immune cell types and the imbalance between anti‐ and pro‐inflammatory mediators associated with obesity lead to chronic inflammation, creating an environment for neoplastic transformation.^256^, ^257^ Tumor cells interact with immune cells, influencing their maturation, differentiation, functional activities, migration, and recruitment to the TME, which may be linked to increased tumor progression and immune escape.^16^, ^236^, ^258^ Individuals with obesity exhibit alterations in the functionality of immune cells, which contribute to the formation of populations with altered activity and possible cancer‐promoting capabilities. Studies have also suggested a potential dual role for certain cell types in processes associated with tumor formation and development.^258^, ^259^ This, combined with a limited understanding of the interactions within the immune cell network, provides an inconclusive picture of the overall impact of the immune cells that populate WAT on CRC.

## 7 CONCLUSIONS

Research to date has shown a significant association between obesity and CRC, both as a risk and prognostic factor. BMI has been identified as a significant risk factor in patients with CRC.^260^, ^261^ This parameter is positively correlated with early‐onset cancer. Younger adults with overweight and obesity (BMI > 25 kg/m^2^) have an approximately 32% and 88% higher risk of CRC, respectively, than healthy individuals of a healthy weight. According to a meta‐analysis included in a previous report issued by the World Cancer Research Fund (WCRF) and the American Institute for Cancer Research (AICR), there is a 5% increase in cancer risk for every 5 units of increase in BMI, with an association stronger than approximately 27 kg/m^2^.^261^, ^262^ The correlation between BMI and CRC development appears to be more complicated, with a trend linked to tumor advancement, among other factors. A significantly reduced BMI (<18.5 kg/m^2^), as well as low muscle index and density, were negative prognostic factors and correlated with a poorer prognosis for patients.^263^, ^264^ However, obesity may be an asset in patients suffering from CRC, where a major reduction in body weight may be associated with a worse prognosis, especially in the later stages of the disease.^260^, ^264^ Clinical studies have indicated that pre‐diagnostic administration of weight loss medications reduces the incidence of CRC.^265^ Furthermore, recent medical data suggest that bariatric surgery may reduce the incidence of CRC in individuals with obesity. The observed effect was independent of the surgical procedure or patient sex.^266^ Although these reports are intriguing, further studies using patient cohorts are essential to confirm these results. Clinical evidence and meta‐analyses emphasize the correlation between BMI and CRC risk, indicating that the BMI of individuals with obesity as risk factor for CRC in the general population.^267^, ^268^, ^269^ Moreover, physical activity and healthy diet may decrease the risk of CRC.^261^, ^270^, ^271^ However, this correlation needs further elucidation, as some data indicate no correlation^272^, ^273^ and point to low BMI as a risk factor for progression and death in metastatic CRC.^274^, ^275^ In addition, the current data suggest a possible role for lifestyle and diet in the overall survival and recurrence of CRC, implying the relevance of these factors for CRC patients with CRC.^276^, ^277^, ^278^ Therefore, it is controversial whether weight loss during tumor progression positively affects patient outcomes.

Current evidence suggests that weight reduction may be linked to a significant downregulation of CRC risk. Studies have demonstrated that weight loss is associated with a reduction in the levels of inflammatory markers such as TGFβ, TNFα, IL‐6, interleukin 18 (IL‐18), MCP‐1, C‐reactive protein (CRP), and leptin, which are known to contribute to CRC development.^1^, ^279^ Additionally, weight loss induces an upregulation in anti‐inflammatory and protective factors like IL‐10 and adiponectin, both implicated in cancer suppression, as well as reduction in the activity of the NLR family pyrin domain containing 3 (NLRP3) inflammasome in patients with type 2 diabetes, a critical mediator of chronic inflammation.^1^, ^280^ This weight loose‐related shift in the secretory profile of Adipose tissue (AT) may play a crucial role in lowering CRC risk.^281^, ^282^ While current data remain somewhat limited, the available research suggests a connection between weight loss and decreased CRC susceptibility indicating that weight loss may confer benefits in reducing cancer risk.^281^, ^283^ Nevertheless, the intricate network of interactions underlying these observations requires thorough investigation to be sufficiently clarified.

**FIGURE 4:**
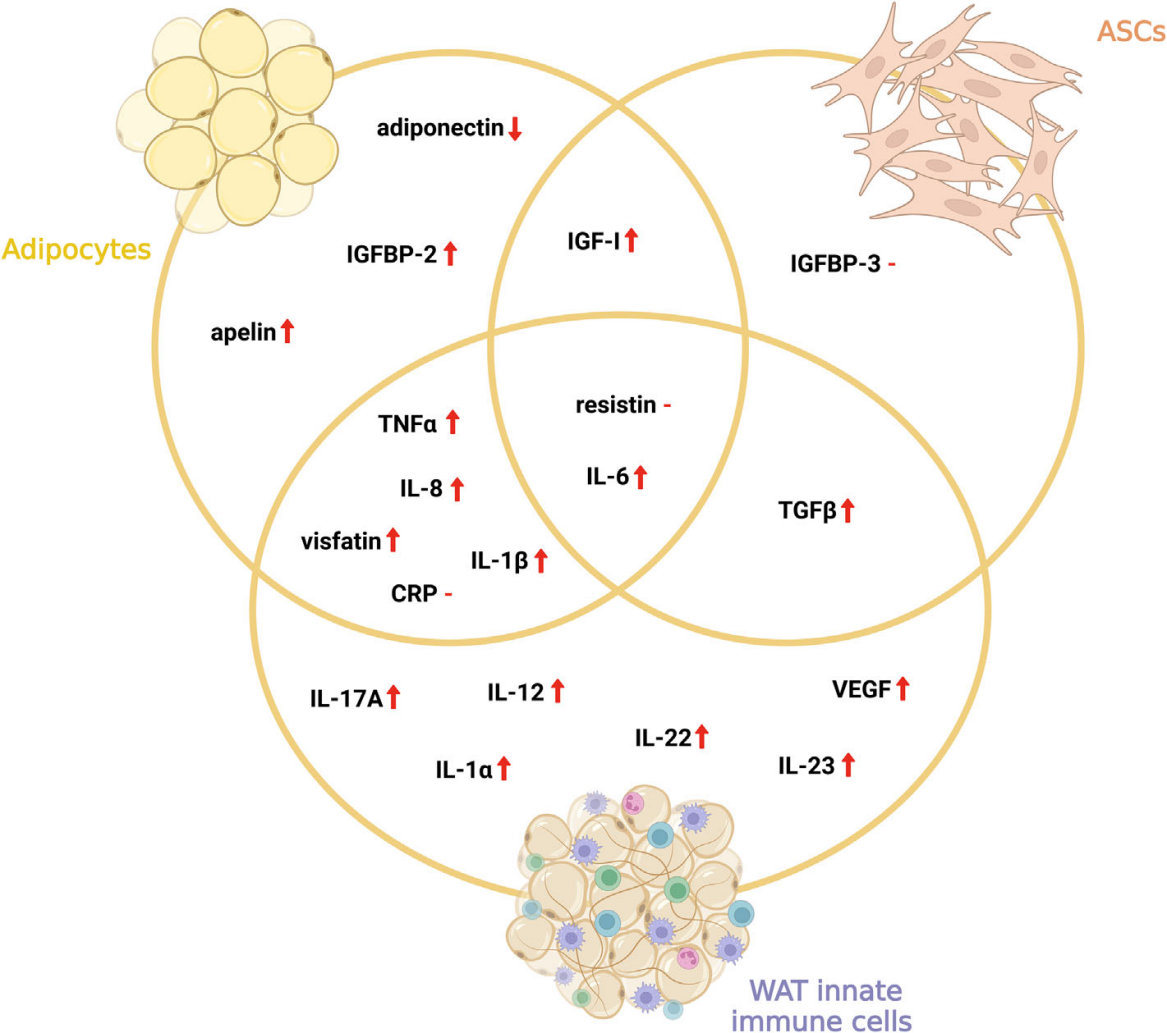
Molecules released by adipose tissue cells and their impact on colorectal cancer progression. Circles mark molecules released by a given cell type. “‐” stands for unclear level, ↑ stands for stimulation of progression, and ↓ stands for inhibition of progression (created with BioRender). AT, adipose tissue; CRP, C reactive protein; IGFBP‐2, insulin‐like growth factor binding protein 2; IGFBP‐3, insulin‐like growth factor binding protein 3; IGF‐I, insulin‐like growth factor I; IL‐12, interleukin 12; IL‐17A, interleukin 17A; IL‐1α, interleukin 1α; IL‐1β, interleukin 1β; IL‐22, interleukin 22; IL‐23, interleukin 23; IL‐6, interleukin 6; IL‐8, interleukin 8; TGFβ, tumor growth factor β; TNFα, tumor necrosis factor α; VEGF, vascular endothelial growth factor. (created with BioRender).

Recent years have brought about a significant expansion of knowledge regarding the molecular basis of CRC development and the involvement of adipose tissue cells in this process (crucial molecules are summarized in Figure 4). Despite the efforts of scientists, CRC remains one of the most serious issues in terms of incidence and mortality. Intensive research has established the view of WAT not only as a fat reservoir but also as an important compartment constituting a source of cells implicated in TME formation. Notably, the involvement of diverse cell types inhabiting the WAT, as highlighted in this review, is critical for understanding the complex web of interactions that shape the formation of an environment supporting CRC progression. They support and modulate, either directly or indirectly, the processes involved in the initiation, enhanced invasiveness, and migration of tumor cells and their potential to maintain stemness characteristics.

A broader perspective on WAT and the cells inhabiting it will provide further insights into the identification of therapeutic targets and the design of new, more specific, and precise anti‐cancer therapies. Therefore, there is an ongoing need to study WAT as a multicellular compartment to obtain a more comprehensive and holistic view of the complex network of interactions within the adipose TME.

## CONFLICT OF INTEREST STATEMENT

The authors declare that they have no conflicts of interest.

## Supporting information


**Table S1.** Comparison of the influence of cytokines released by cells typically inhabiting adipose tissue, their impact on colorectal cancer progression and fluctuations in level under obesity condition. Abbreviations: AT‐ adipose tissue, CRC‐ colorectal cancer, CRP‐ C reactive protein, DCs‐ Dendritic cells, EMT‐ epidermal to mesenchymal transition, NK‐ natural killer, SAT‐ subcutaneous adipose tissue, TGFβ – tumor growth factor β, TNFα – tumor necrosis factor α, TNM‐ tumor node Metastasis, VAT‐visceral adipose tissue, VEGF – vascular endothelial growth factor, WAT‐white adipose tissue↑ higher level, ↓ lower level, ‐ no significant differences.
