# Supplementary material for: The role of adipogenic niche resident cells in colorectal cancer progression in relation to obesity
Source: Obes Rev. 2025 Jan 6;26(4):e13873. doi: 10.1111/obr.13873 (PMC11884973; doi:10.1111/obr.13873)
Supplement: Supplementary file 1 — Table S1. Comparison of the influence of cytokines released by cells typically inhabiting adipose tissue, their impact on colorectal cancer progression and fluctuations in level under obesity condition. Abbreviations: AT‐ adipose tissue, CRC‐ colorectal cancer, CRP‐ C reactive protein, DCs‐ Dendritic cells, EMT‐ epidermal to mesenchymal transition, NK‐ natural killer, SAT‐ subcutaneous adipose tissue, TGFβ – tumor growth factor β, TNFα – tumor necrosis factor α, TNM‐ tumor node Metastasis, VAT‐visceral adipose tissue, VEGF – vascular endothelial growth factor, WAT‐white adipose tissue↑ higher level, ↓ lower level, ‐ no significant differences. [file OBR-26-e13873-s001.pdf]

The role of adipogenic niche resident cells in colorectal cancer progression in relation to obesity

Running title: Colorectal cancer and white adipose tissue: the impact of obesity

Mikołaj Domagalski, Joanna Olszańska, Katarzyna Pietraszek-Gremplewicz[#], Dorota Nowak[#]

Department of Cell Pathology, Faculty of Biotechnology, University of Wroclaw, Joliot-Curie 14a, 50-383, Wroclaw, Poland

# Correspondence

Katarzyna Pietraszek-Gremplewicz

Email: katarzyna.pietraszek-gremplewicz@uwr.edu.pl

Dorota Nowak

Email: dorota.nowak@uwr.edu.pl

Table S1. Comparison of the influence of cytokines released by cells typically inhabiting adipose tissue, their impact on colorectal cancer progression and fluctuations in level under obesity condition. Abbreviations: AT- adipose tissue, CRC- colorectal cancer, CRP- C reactive protein, DCs- Dendritic cells, EMT- epidermal to mesenchymal transition, NK- natural killer, SAT- subcutaneous adipose tissue, TGFβ – tumor growth factor β, TNFα – tumor necrosis factor α, TNM- tumor node Metastasis, VAT-visceral adipose tissue, VEGF – vascular endothelial growth factor, WAT-white adipose tissue↑ higher level, ↓ lower level, - no significant differences.

Molecule	Expressing cells from AT	Importance in CRC	Level under obesity
**TNFα**	Macrophages, mast cells [1];<br>Adipocytes [2];<br>Natural killers, T cells [1,3];<br>B cells [3]	Promotion of CRC cell migration [4]	↑ in serum [5]<br>↑ in adipose tissue of woman with obesity [6]
**IL-1α**	Myeloid cells [7]	Up-regulation of angiogenesis [8]	
**IL-1β**	Macrophages, monocytes, dendritic cells (DCs) [9];<br>Adipocytes [10]	Induction of CRC cells growth [11];<br>Promotion of EMT and stem cell development in colon cancer cells [12]	↑ mRNA in subcutaneous AT (SAT) [13]
**IL-6**	Macrophages [1,14];<br>Monocytes, T- cells, B-lymphocytes [1];	Protection of colon cancer cells from apoptosis [17];<br>Promotion the tumor formation, growth [18] and CRC cell proliferation in vitro [19];	↑ in serum of patients with visceral adiposity [5]<br>↑ mRNA and protein level in SAT [21,22]

	Adipocytes [2];<br>Adipose stromal cells [15];<br>Mast cells [16]	Association between serum concentration in patients and the progression, histological grade, tumor size, shorter survival periods and bowel wall invasion of CRC [20]	
**IL-8**	Adipocytes [2];<br>Neutrophils, macrophages [22]	Promotion of stemness of CRC [23];<br>Stimulation of proliferation, migration, invasion and chemoresistance of CRC cells [24]	↑ in serum [25]<br>↑ in plasma [26]<br>↑ mRNA in WAT [22]
**IL-12**	Macrophages [14];<br>DCs [27]	Antitumor activity in mice [28] [29] and humans [30]	↑ in serum [31]<br>- in plasma of women with obesity [32]
**IL-17A**	T – helper cells, γδT cells, natural killer T cells, neutrophils, mast cells [33]	Promotion of angiogenesis [34];<br>Stimulation of CRC cell migration [35]	↑ in plasma of women with obesity [32]<br>↓ in serum and ↑ mRNA in AT of morbidity women with obesity [21]
**IL-22**	T cells, T- helper cells, γδT cells, NK cells ;<br>Neutrophils [36];	High level in the CRC microenvironment promotes tumor growth, metastasis and inhibits apoptosis [37];<br>Stimulation of tumor growth [38], promotion the proliferation, migration, invasion, chemotherapy resistance and stemness of colon cancer [39]	- in serum [40]

**IL-23**	DCs, monocytes,macrophages [41]; Neutrophils [42]	Promotion of early carcinogenesis, tumor growth and progression of colorectal tumors [42]	↑ in plasma of women with obesity [32]
**CRP**	Macrophages, lymphocytes, adipocytes [43]	No correlation between circulating CRP and CRC development [44];<br>Higher serum level in CRC patients than control subjects, and positive correlation with TNM classification [45]	↑ in serum [5]
**TGFβ**	Macrophages, preadipocytes, adipose stromal cells [46]	Stimulation of tumor metastasis [47] and EMT in vitro [48];<br>Inhibition of angiogenesis in vivo [49];<br>Mediator in resistance to apoptosis [50]	↑ in AT [51]<br>↑ in serum [52]<br>↑ in plasma [53]<br>↓ in plasma of women with obesity [54]

1. Klampfer, L. Cytokines, Inflammation and Colon Cancer. *Curr Cancer Drug Targets* **11**, 451–464 (2011).

2. Fain, J. N. Release of Interleukins and Other Inflammatory Cytokines by Human Adipose Tissue Is Enhanced in Obesity and Primarily due to the Nonfat Cells. in *Vitamins & Hormones* vol. 74 443–477 (Academic Press, 2006).

3. Aggarwal, B. B. Signalling pathways of the TNF superfamily: A double-edged sword. *Nat Rev Immunol* **3**, 745–756 (2003).

4. Kobelt, D., Zhang, C., Clayton-lucey, I. A. & Glauben, R. Pro-inflammatory TNF- α and IFN- γ Promote Tumor Growth and Metastasis via Induction of MACC1. **11**, 1–15 (2020).

5. Park, H. S., Park, J. Y. & Yu, R. Relationship of obesity and visceral adiposity with serum concentrations of CRP, TNF-α and IL-6. *Diabetes Res Clin Pract* **69**, 29–35 (2005).

6. Fain, J. N., Bahouth, S. W. & Madan, A. K. TNFα release by the nonfat cells of human adipose tissue. *Int J Obes* **28**, 616–622 (2004).

7. Cavalli, G. *et al.* Interleukin 1α: a comprehensive review on the role of IL-1α in the pathogenesis and treatment of autoimmune and inflammatory diseases. *Autoimmun Rev* **20**, 102763 (2021).

8. Matsuo, Y. *et al.* IL-1 a Secreted by Colon Cancer Cells Enhances Angiogenesis : The Relationship Between IL-1 a Release and Tumor Cells ' Potential for Liver Metastasis. 361–367 (2009) doi:10.1002/jso.21245.

9. Fields, J. K., Günther, S. & Sundberg, E. J. Structural Basis of IL-1 Family Cytokine Signaling. *Front Immunol* **10**, 1–20 (2019).

10. Pellegrinelli, V. *et al.* Human Adipocytes Induce In fl ammation and Atrophy in Muscle Cells During Obesity. *Diabetes* **64**, 3121–3134 (2015).

11. Kaler, P., Augenlicht, L. & Klampfer, L. Macrophage-derived IL-1 b stimulates Wnt signaling and growth of colon cancer cells : a crosstalk interrupted by vitamin D 3. *Oncogene* **28**, 3892–3902 (2009).

12. Li, Y., Wang, L., Pappan, L., Galliher-Beckley, A. & Shi, J. IL-1β promotes stemness and invasiveness of colon cancer cells through Zeb1 activation. *Mol Cancer* **11**, 1–13 (2012).

13. Moschen, A. R. *et al.* Adipose and liver expression of interleukin (IL)-1 family members in morbid obesity and effects of weight loss. *Molecular Medicine* **17**, 840–845 (2011).

14. Scheurlen, K. M., Billeter, A. T., O'Brien, S. J. & Galandiuk, S. Metabolic dysfunction and early-onset colorectal cancer – how macrophages build the bridge. *Cancer Med* **9**, 6679–6693 (2020).

15. Di Franco, S. *et al.* Adipose stem cell niche reprograms the colorectal cancer stem cell metastatic machinery. *Nat Commun* **12**, (2021).

16. Unamuno, X., Gema, F. & Victoria, C. Adipose tissue. in *Encyclopedia of Endocrine Diseases* 370–384 (Elsevier, 2018). doi:10.1016/B978-0-12-801238-3.65163-2.

17. Yuan, H., Liddle, F. J., Mahajan, S. & Frank, D. A. IL-6-induced survival of colorectal carcinoma cells is inhibited by butyrate through down-regulation of the IL-6 receptor. *Carcinogenesis* **25**, 2247–2255 (2004).

18. Wei, H.-J. *et al. Adipose-Derived Stem Cells Promote Tumor Initiation and Accelerate Tumor Growth by Interleukin-6 Production*. *Oncotarget* vol. 6 www.impactjournals.com/oncotarget/ (2015).

19. Fang, X. *et al.* CRH promotes human colon cancer cell proliferation via IL-6 / JAK2 / STAT3 signaling pathway and VEGF-induced tumor angiogenesis. *Mol Carcinog* (2017) doi:10.1002/mc.22691.

20. Guo, Y., Xu, F., Lu, T., Duan, Z. & Zhang, Z. Interleukin-6 signaling pathway in targeted therapy for cancer. *Cancer Treat Rev* **38**, 904–910 (2012).

21. Zapata-Gonzalez, F. *et al.* Interleukin-17A gene expression in morbidly obese women. *Int J Mol Sci* **16**, 17469–17481 (2015).

22. Rouault, C. *et al.* Roles of Chemokine Ligand-2 (CXCL2) and Neutrophils in Influencing Endothelial Cell Function and Inflammation of Human Adipose Tissue. *Endocrinology* **154**, 1069–1079 (2013).

23. Ma, X. *et al.* Mesenchymal stem cells maintain the stemness of colon cancer stem cells via interleukin-8/mitogen-activated protein kinase signaling pathway. *Exp Biol Med* **245**, 562–575 (2020).

24. Ning, Y. *et al.* Interleukin-8 is associated with proliferation, migration, angiogenesis and chemosensitivity in vitro and in vivo in colon cancer cell line models. **128**, 2038–2049 (2012).

25. Kim, C. *et al.* Circulating levels of MCP-1 and IL-8 are elevated in human obese subjects and associated with obesity- related parameters. *Int J Obes* **30**, 1347–1355 (2006).

26. Straczkowski, M. *et al.* Plasma Interleukin-8 Concentrations Are Increased in Obese Subjects and Related to Fat Mass and Tumor Necrosis Factor-α System. *J Clin Endocrinol Metab* **87**, 4602–4606 (2002).

27. Aass, K. R., Kastnes, M. H. & Standal, T. Molecular interactions and functions of IL-32. *J Leukoc Biol* (2020) doi:10.1002/JLB.3MR0620-550R.

28. Gambotto, A. *et al.* Induction of antitumor immunity by direct intratumoral injection of a recombinant adenovirus vector expressing interleukin-12. *Cancer Gene Ther* **6**, 45–53 (1999).

29. Halin, C. *et al.* Enhancement of the antitumor activity of interleukin-12 by targeted delivery to neovasculature. *Nat Biotechnol* **20**, 264–269 (2002).

30. O'Hara, R. J. *et al.* Advanced colorectal cancer is associated with impaired interleukin 12 and enhanced interleukin 10 production. *Clinical Cancer Research* **4**, 1943–1948 (1998).

31. Schmidt, F. M. *et al.* Inflammatory cytokines in general and central obesity and modulating effects of physical Activity. *PLoS One* **10**, (2015).

32. Sumarac-Dumanovic, M. *et al.* Increased activity of interleukin-23/interleukin-17 proinflammatory axis in obese women. *Int J Obes* **33**, 151–156 (2009).

33. Ge, Y., Huang, M. & Yao, Y. Biology of Interleukin-17 and Its Pathophysiological Significance in Sepsis. **11**, 1–13 (2020).

34. Numasaki, M. *et al.* Interleukin-17 promotes angiogenesis and tumor growth. *Blood* **101**, 2620–2627 (2003).

35. Chin, C. *et al.* Interleukin-17 Induces CC Chemokine Receptor 6 Expression and Cell Migration in Colorectal Cancer Cells. *J Cell Physiol* 1430–1437 (2014) doi:10.1002/jcp.24796.

36. Zenewicz, L. A. IL-22: There Is a Gap in Our Knowledge. *Immunohorizons* **2**, 198–207 (2018).

37. Jiang, R. *et al.* IL-22 is related to development of human colon cancer by activation of STAT3. *BMC Cancer* **13**, (2013).

38. Perez, L. G. *et al.* TGF-β signaling in Th17 cells promotes IL-22 production and colitis-associated colon cancer. *Nat Commun* **11**, (2020).

39. Xi, X. *et al.* Interleukin-22 promotes PD-L1 expression via STAT3 in colon cancer cells. *Oncol Lett* **22**, 1–5 (2021).

40. Zhao, R. *et al.* Elevated peripheral frequencies of th22 cells: A novel potent participant in obesity and type 2 diabetes. *PLoS One* **9**, (2014).

41. Egeberg, A., Gisondi, P., Carrascosa, J. M., Warren, R. B. & Mrowietz, U. The role of the interleukin-23/Th17 pathway in cardiometabolic comorbidity associated with psoriasis. *Journal of the European Academy of Dermatology and Venereology* **34**, 1695–1706 (2020).

42. Neurath, M. F. IL-23 in inflammatory bowel diseases and colon cancer. *Cytokine Growth Factor Rev* **45**, 1–8 (2019).

43. Sproston, N. R. & Ashworth, J. J. Role of C-reactive protein at sites of inflammation and infection. *Front Immunol* **9**, 1–11 (2018).

44. Nimptsch, K. *et al.* Pre-diagnostic C-reactive protein concentrations, CRP genetic variation and mortality among individuals with colorectal cancer in Western European populations. *BMC Cancer* **22**, 1–13 (2022).

45. Elessawi, D. F., Alkady, M. M. & Ibrahim, I. M. Diagnostic and prognostic value of serum IL-23 in colorectal cancer. *Arab Journal of Gastroenterology* **20**, 65–68 (2019).

46. Lee, M. Transforming growth factor beta superfamily regulation of adipose tissue biology in obesity. *BBA - Molecular Basis of Disease* **1864**, 1160–1171 (2018).

47. Wang, X. *et al.* Oxymatrine inhibits the migration of human colorectal carcinoma RKO cells via inhibition of PAI-1 and the TGF- β 1 / Smad signaling pathway. *Oncol Rep* **37**, 747–753 (2017).

48. Lu, C., Yang, Z., Yu, D., Lin, J. & Cai, W. RUNX1 regulates TGF-β induced migration and EMT in colorectal cancer. *Pathol Res Pract* **216**, 153142 (2020).

49. Geng, L., Chaudhuri, A., Talmon, G., Wisecarver, J. L. & Wang, J. TGF-Beta Suppresses VEGFA-Mediated Angiogenesis in Colon Cancer Metastasis. **8**, 1–8 (2013).

50. Oulay, J. B. *et al.* SMAD7 IS A PROGNOSTIC MARKER IN PATIENTS WITH COLORECTAL CANCER. **449**, 446–449 (2003).

51. Fain, J. N., Tichansky, D. S. & Madan, A. K. Transforming Growth Factor β1 release by human adipose tissue is enhanced in obesity. *Metabolism* **54**, 1546–1551 (2005).

52. Lin, Y. *et al.* Variations in serum transforming growth factor-β1 levels with gender, age and lifestyle factors of healthy Japanese adults. *Dis Markers* **27**, 23–28 (2009).

53. Porreca, E. *et al.* Transforming growth factor-β1 levels in hypertensive patients: Association with body mass index and leptin. *Am J Hypertens* **15**, 759–765 (2002).

54. Corica, F. *et al.* Reduced plasma concectrations of transforming growth factor β1 (TGF-β1) in obese women. *Int J Obes* **21**, 704–707 (1997).
